# Healthcare educators’ perspectives on IPE implementation at a Saudi university: a qualitative study

**DOI:** 10.3389/fmed.2026.1703063

**Published:** 2026-01-27

**Authors:** Mohra Aladwani

**Affiliations:** Department of Clinical Pharmacy, College of Pharmacy, Taif University, Taif, Saudi Arabia

**Keywords:** educators, faculty, healthcare education, higher education, interprofessional education (IPE), Saudi Arabia

## Abstract

**Background:**

Interprofessional education is one of the pedagogies recommended to help enhance the knowledge and skills of future healthcare workers. Educators are important stakeholders in IPE implementation. This study aimed to explore educators’ views on IPE implementation at Taif University.

**Methods:**

Healthcare educators and leaders at the university participated in a qualitative study conducted between October 2024 and February 2025. Semi-structured interviews were conducted via Zoom. Data collection and analysis were guided by the SWOC framework (strengths, weaknesses, opportunities, and challenges). Thematic analysis was conducted to analyze the data.

**Results:**

A total of 21 educators from healthcare programs participated in the study. Educators with prior exposure to IPE, the location of some of the colleges, and the presence of a simulation center were perceived as strengths. Opportunities were identified in improving students’ attitudes and skills within a collaborative work environment, and in enhancing collaboration with staff from various programs and clinicians from hospitals. Logistical barriers, staff resistance, bureaucratic approval processes, hierarchies, and lack of a university hospital were perceived as weaknesses and challenges that might hinder implementation efforts.

**Conclusion:**

This exploratory study provides insights into the views of educators and leaders about IPE implementation at Taif University. The overwhelming majority of participants had positive views on IPE integration into healthcare programs. Educators with prior exposure to IPE can advocate for IPE and help raise awareness of IPE among university staff and students. Decision-makers involved in implementing IPE can use these findings and make efforts to address the challenges and weaknesses identified.

## Introduction

Interprofessional education (IPE) has been implemented in healthcare education since the 1980s in different healthcare schools in the United Kingdom and the United States. The aim of IPE is to improve healthcare learners’ attitudes, knowledge, and skills toward collaborative practice (1). IPE occurs when students from two or more professions learn with, from, and about each other to improve collaboration and the quality of care (2). Healthcare collaborative practice has been shown to improve overall care quality, patients’ clinical outcomes, and healthcare workers’ satisfaction in different settings (3). Early studies have shown that IPE can positively enhance healthcare learners’ attitudes and knowledge regarding other professions and teamwork (4, 5). For years, evidence regarding the impact of IPE on learners’ skills has been lacking due to an overreliance on quantitative methods for evaluating IPE impact (6, 7). However, evidence from a recent review of 94 studies found strong evidence indicating a positive relationship between IPE and various key quality measures, including hospital stay, medical errors, patient satisfaction, patient or caregiver education, and mortality rate (8). Thus, the call for incorporating IPE within healthcare undergraduate education to improve future healthcare workers’ attitudes and skills has become stronger.

For decades, healthcare education has been offered in silos, where each discipline or program has its own learning goals and competencies to achieve (9). Transforming into new integrated education approaches such as IPE might hold uncertainty, challenges and in some cases a high degree of resistance (10). Thus, the involvement of different stakeholder groups within higher education, including university leaders, educators, administrators, and researchers, is needed to enable the inception of new teaching pedagogies. Healthcare educators play a significant role in this transformation process. Successful implementation of IPE requires enthusiastic, cooperative, and committed educators (11, 12).

Several studies have explored educators’ views and attitudes toward IPE (13, 14). In a study that recruited educators across the US, researchers examined the positive and negative factors influencing educators’ engagement with IPE implementation in healthcare schools. Among the positive factors reported were students benefits, improved patient care, and healthcare teamwork, whereas the negative factors included scheduling conflicts, territorial issues, and attitudes between and toward other disciplines. Several barriers to embedding IPE into healthcare curricula have been identified in a review by Lawlis et al. (15), including a lack of collaborative attitudes between disciplines, a lack of understanding of IPE, a lack of perceived value, and a lack of faculty rewards (15).

In Arab countries, IPE is still emerging and has been implemented in only some universities over the past decade (16, 17). Several studies have been published, most of which have measured students’ attitudes and perceptions of IPE (18, 19). In terms of healthcare educators’ perspectives, little has been explored and published. El-Awaisi et al., explored pharmacy educators’ attitudes toward introducing IPE into healthcare schools across several Arab countries and reported overall positive attitudes (20). In a recent study by Alruthia et al. (21), healthcare academics’ views were explored regarding IPE implementation at King Saud University in Saudi Arabia (21). The opportunities reported in the study included better education outcomes, incentives, and better utilization of human and information resources at the university. Physicians’ egos, resistance to change, and difficulties in designing IPE courses were among the obstacles identified.

Taif University is a Saudi government university in the Western region that was founded in 1980. In 2024, the university had over forty-two thousand students and four thousand staff. It offers undergraduate and postgraduate degrees in different specialties, including education and arts, science and engineering, and healthcare specialties. Since IPE is on the agenda of the university’s leaders, it is important to explore the views of different stakeholders, including students, educators, and leaders from healthcare programs at Taif University. In a previous study, the author and a research team explored the readiness of healthcare students toward IPE at the university (22). Students from healthcare programs showed positive attitudes toward and high readiness levels for IPE. Thus, based on the recommendations of the previous study (22) to further explore the topic with other stakeholder groups, the targeted groups in this study were healthcare educators and leaders at Taif University.

The SWOC tool (strengths, weaknesses, opportunities, and challenges) is a widely used strategy to guide implementation plans in different settings (23). Therefore, this study aimed to identify the strengths, weaknesses, opportunities, and challenges related to IPE implementation at Taif University from the perspective of stakeholder groups.

## Materials and methods

To meet the purpose of the study, a qualitative approach using semi-structured interviews was adopted. Individual interviews are indicated to obtain rich and deep insights from the participant regarding the topic of interest (24). Purposeful sampling was used to recruit healthcare educators involved in teaching undergraduate courses across healthcare colleges at Taif University, including medicine, pharmacy, dentistry, nursing, and applied medical sciences.

Potential participants were recruited by sending invitations via email and WhatsApp, which are both used for work communications at the university. Furthermore, snowball sampling was used, where participants were asked to invite their colleagues to the study. Zoom was used to conduct interviews, and at the start of each interview, the participants verbally consented to participate and agreed for the interviews to be audio recorded. The topic guide for the interviews was designed considering the main components of the SWOC tool and relevant previous studies (20, 21). The topic guide was tested with two healthcare educators from nursing and pharmacy programs. The guide was then edited for accuracy and clarity based on their suggestions. The study was approved by the Institutional Review Board at Taif University (Application number: HAO-02-T-105).

Participants were given the choice to choose their preferred language throughout the interview, either Arabic or English. Audio recordings of the interviews were uploaded to TurboScribe application to be transcribed verbatim. Arabic interviews were translated into English using the translation feature of the same application. The audio recordings and transcripts were reviewed by the author and a research assistant for accuracy of the transcribing. Additionally, the author reviewed the translated transcripts to ensure the accuracy of the translation by TurboScribe. Data collection continued until data saturation was achieved (25).

Thematic analysis was used to analyze the data, applying the six-phase method suggested by Braun and Clarke: familiarization with the data, generation of codes, identification of themes, review of themes, categorization of themes, and writing of the report (26). The author familiarized herself with the data then created an initial codes list. To enhance the trustworthiness of the analysis, a research assistant with experience in qualitative research separately analyzed the transcripts and created a list of initial codes. The initial codes from both researchers were then compared across all transcripts and finalized upon discussion. Similar codes were then categorized into major themes in relation to the main components of the SWOC tool: strengths, weaknesses, opportunities, and challenges.

## Results

A total of 21 educators from different healthcare programs at Taif University were interviewed for this study. Data collection was conducted from October 2024 to February 2025. The average time for the interviews was 40 min. Table 1 summarizes the participants’ demographic characteristics. The numbers of males and females were 10 and 11, respectively and were a mix from all healthcare colleges. Most of the participants were assistant professors, except for two who were associate professors and one who was an assistant lecturer. Seven participants held leadership positions at different levels, including deans, vice-deans, and heads of departments. However, the exact position was not disclosed to avoid compromising participants’ anonymity.

**TABLE 1:** Characteristics of the participants (*n* = 21).

Characteristic	Number	Percentages
**Gender**		
Female	11	52.38
Male	10	47.62
**College**		
Applied medical sciences	3	14.29
Medicine	6	28.57
Pharmacy	7	33.33
Nursing	5	23.81
**Academic rank**		
Assistant Professor	18	85.714
Associate professor	2	9.524
Assistant lecturer	1	4.762
**Leadership**		
Yes	7	33.33
No	14	66.67

The data were categorized into themes considering the framework applied, strengths, weaknesses, opportunities, and challenges. Table 2 summarizes the main findings of each category.

**TABLE 2:** The main findings catgorized in accordance with the SWOC framework.

Strengths	Weaknesses
• Educators with prior exposure to IPE • Location of colleges. • The presence of a simulation center.	• Logistical barriers (e.g., IPE for medicine and nursing). • Lack of a university hospital. • Lack of adequate resources for IPE teaching. • Dropout/transfer of students from Applied Medical Sciences.
**Opportunities**	**Challenges**
• Benefits of IPE for students. • Enhance the collaboration between educators. • Enhance learning and research outputs. • Enhance collaboration with clinicians from hospitals. • IPE activities in community services.	• Bureaucratic approval process • Staff resistance • Professional battles and hierarchies • Staff ability to facilitate IPE

### Strengths

Participants highlighted some strengths of IPE implementation at Taif University, including educators with prior exposure to IPE, the location of some of the colleges, and the presence of a simulation center.

Some of the educators interviewed were not aware of the term interprofessional education; therefore, the researcher explained its meaning and provided examples from the literature at the beginning of the interviews. Considering the purpose of IPE, educators had positive views about its implementation at the university.

“Interviewer: Have you ever heard the term interprofessional education before now?

*Educator: To be honest with you, I have not heard about that before. But I think if we implement it, it will increase the knowledge of the student about other professions. In the future, the student will know how to refer to other departments or other teams.” M7, Pharmacy.*

Other educators recalled experiences of learning events similar to IPE as part of their postgraduate education. Educators with previous exposure to IPE were very keen to see IPE implemented at the university.

*“I was first introduced to Interprofessional Education (IPE) during my Master’s studies in the United States, where it was an integral part of the educational system. Experiencing IPE as a student provided me with a valuable perspective and a deeper understanding of this approach” F11, Nursing.*

*“I think like a good point of Taif University that most of the staff working here at Taif University. I think they passed through similar experience. They might hear or they might experience it through the universities that the staff have already studied in abroad.”F3, Applied Medical Sciences.*

The location of some colleges was perceived as a strength. The buildings of medicine, pharmacy, and dentistry are adjacent to each other, so students and staff can easily move between the buildings. Participants also mentioned the proximity of nursing and applied medical sciences, which can facilitate IPE sessions for students in these programs in the future.

*“The proximity of buildings, such as the medicine and pharmacy departments being located near each other, makes it convenient to organize and conduct IPE sessions” F7, Pharmacy.*

Among the perceived strengths is the availability of a simulation center at Taif University. Some educators expressed their views on how IPE should be taught in the form of skills-based learning. Therefore, they thought IPE should be designed and delivered through simulation experiences and suggested utilizing the simulation center.

*“We do actually get the luxury here in Taif University with the simulation center. I see it [IPE] as a training during internship or later years with simulation” M1, Medicine.*

### Weaknesses

The identified weaknesses based on participants’ views included logistical issues, the lack of a university hospital, lack of adequate resources, and the high drop-out rate in applied medical sciences students.

Although the location was mentioned earlier as a strength, participants anticipated difficulties in scheduling IPE events for some programs due to logistical considerations, such as students from medicine and nursing or pharmacy and physical therapy or laboratory sciences. Educators explained that the nursing and applied medical sciences colleges are located on a campus that is 30 min away by car from the medicine and pharmacy colleges.

*“So I feel like for College of Medicine and College of Pharmacy, it’s okay. But College of Physical Therapy, it’s far away from College of Medicine and Pharmacy. And it will be difficult for students to come together.” M7, Pharmacy.*

*“So students from medicine and pharmacy on the same area, like the buildings are close to each other. But when we think about nursing, their college is far away from here.”M2, Medicine.*

To overcome this issue, some suggested having a special center dedicated to IPE built in a place that is known and convenient for all programs.

*“So if we just do it, for example, like a shared place in the middle, like different design for the classroom. They have to be round tables, small tables for discussion.”*

Educators believed that the presence of a university hospital is necessary for IPE to be integrated into the clinical training experiences of students from different colleges. Thus, the lack of a university hospital was perceived as a disadvantage.

*“If we’re thinking of like collecting the students in one place, for example, like a university hospital, we still don’t have this.” F3, Applied Medical Sciences.*

“If IPE can be delivered in a skills-based lab, it can be offered in a university hospital. However, the lack of a university hospital makes it difficult to do it.”M6, Pharmacy.

Some educators suggested using resources available at the collaborative hospitals in the region, including hospitals of the Ministry of Health or the Ministry of Defense.

*“We can do it [*IPE*] in one of the hospital, for example, the military or the Ministry of health hospitals that we all collaborate with for our students training” M1, Medicine.*

Another weakness reported by educators was the lack of adequate resources to support the efficient delivery of IPE. The lack of large rooms to accommodate the large number of students was mentioned. In addition, classes equipped especially for small group discussions were among the required resources highlighted, as some educators believed that the core of IPE is for students to have discussions.

*“For example, having a place or a lecture big enough to have everyone, or even like small groups in small rooms, but then a large number of rooms will be needed. Because in pharmacy, we have more than 200 in each year.” M4, Pharmacy.*

*“It’s complicated in my classrooms, because only this year I started implementing group discussion. The chairs are actually fixed on the floor, so you cannot allocate them into circles or something like that.” F1, Applied Medical Sciences.*

Another issue that was exclusively reported by educators from the College of Medical Sciences was the high dropout rate/transfer of students. According to the educators, a high number of students in this college dropout or transfer to other programs such as nursing, medicine, or pharmacy. Some educators at these colleges were worried that introducing students to peers from other programs in IPE might exacerbate the existing issue, particularly if this exposure occurred before their professional identities had fully developed.

*“We have a very big problem, because most of the students still leave out of the program, and they transfer to other departments, mainly nursing.” F1, Applied Medical Sciences.*

*“Of my very close experience, I would say students sometimes at the college of applied sciences, if they are learning from other departments or learning about other departments, they tend to hate their own profession, so I don’t agree with like exposing them to other departments or other professions before they are ready and mature enough to understand how they could contribute with their own profession in the field.” F2, Applied Medical Sciences.*

### Opportunities

Educators thought that IPE implementation could create several opportunities, including benefits for students, improved collaboration among educators across healthcare colleges, enhanced collaboration with clinical staff from hospitals, and applying IPE in community-based activities.

All participants, without exception, were supportive of implementing IPE and were keen about its potential benefits for students. Some of the benefits mentioned included increased knowledge about other healthcare professionals, enhanced mutual respect, enhanced knowledge about the patient referral process, and improved communication and teamwork skills.

*“It* [IPE] *will prepare our students to imagine how the real practice is going to be. So they’re going to deal with physicians, with nurses, with other health care providers. It’s going to promote the patient centered care.” M5, Pharmacy.*

*“I think this IPE may help the students to develop essential skills such as teamwork, communication, problem solving and I think all of these are vital in real-world healthcare setting.” M3, Medicine*

Some participants thought that the benefits of IPE would go beyond students, as they believed that IPE would encourage more collaboration with staff from different programs. In addition, some educators thought that promoting IPE through collaborative research projects between educators from different programs would enhance the university’s research output.

*“It* [IPE] *also encourages the exchange of knowledge and best practices, and opens the door to interdisciplinary research opportunities that can elevate the quality of academic output.”F11, Nursing*

*“So if I am in the place of someone who is leading the university or leading a department, I would definitely promote interprofessional education through research and through teaching and this would definitely improve the research” F2, Applied Medical Sciences.*

Other educators thought that implementing IPE could create opportunities for collaboration with hospital staff. Based on their experience, it was believed that clinical staff from hospitals were qualified to be involved in the design and delivery of IPE. It was suggested that efforts from academic educators and hospital staff, in a complementary approach, can help create meaningful learning experiences that mimic real-life scenarios that graduates might encounter in the future.

*“Maybe you can like join some staff from hospitals. I feel it’s going to be better than the college staff. And that’s because if you have both of them, it will be better. So one of them like start the syllabus at the university and the other continue with the cases and the scenarios in the hospitals.” M7, Pharmacy.*

*“We can collaborate with the hospitals. Not only with the doctors but also with the nursing. I imagine they will introduce it* [IPE] *very well because they interact every day with professionals and patients in the hospital” F5, Medicine.*

Educators highlighted the opportunities for healthcare students to work together in community-based activities rather than in lecture-based activities. Educators thought that applying IPE to these activities could create meaningful collaborative experiences for students from different programs. Such activities would allow for the direct application of theoretical knowledge in practical settings and live interactions with people and patients.

*“Another idea for activities to be involved together is community-based projects. Usually it’s conducted for each school. And some activities are even duplicated for each school. So working together and using the expertise and the knowledge of all professions within the same initiatives would be better.” M4, Pharmacy.*

*“If I’m planning for an outside activity like a community service activity, I’m only restricted to have students from laboratory science. Why not nursing? Why not radiation? Why not physiotherapy?” F1, Applied Medical Sciences.*

### Challenges

There were several challenges participants identified, including lengthy bureaucratic approval processes, resistance from staff, professional battles and hierarchies and the ability of staff to facilitate IPE.

Educators reported that the approval process for incorporating IPE into healthcare programs might not be straightforward. From previous experience, educators explained that introducing a new course usually requires submitting a proposal first to the heads of departments, then to the college administration, and then to the university deanship of development and quality.

*“Some of the barriers I think it could be applying the process itself because applying for courses in medical colleges takes time for approval, starting from the department to the college and after that, to the university.” F9, Nursing.*

Participants expressed concerns regarding IPE acceptance by staff at healthcare colleges. Busy schedules, teaching duties, research tasks, and administrative activities were mentioned as reasons that might potentially hold back staff from being engaged in IPE implementation. To encourage staff, educators suggested several measures, including reducing teaching loads, offering financial incentives, presenting appreciation award, and considering IPE involvement in promotions.

*“We are suffering from the low number of doctors. So if we put more effort and more tasks for them, I don’t know if they will accept it or not. So we have to encourage them first.” F5, Medicine.*

*“I think it will be difficult to convince staff to join these sessions. So they need to get something out of these sessions, either a recognition, for example, volunteering certificate, or financial incentives, or accredited hours in their schedule.” M4, Pharmacy.*

Turf battles were seen as a potential challenge, as some educators believed that competing for power might be an issue at the early stages of IPE planning. They described examples of potential conflicts where some staff might want to exert more control over the design, content, and delivery process of IPE.

*“If you introduce this designing for multi-specialty, every one of them, they want to take the lead. They want to put more. It is not about who wants to work. It is about who works more. So, this will be a little bit sensitive” F5, Medicine.*

Some thought that such conflicts might be more likely to occur if staff from the Medicine program were involved. The issue of hierarchies was believed to still exist, where medical staff usually took the lead. These views were shared by medical educators and educators from other programs.

*“When we try to teach in medical rounds in the hospital, sometime the consultant dominates the session. And what happens is the head nurse does not like participate a lot when she’s supposed to. So I guess it will be the same between educational members in the university. I don’t know why medical usually dominate.” M2, Medicine.*

*“If you will introduce this in the medicine, try to give the lead for the medicine. So, they will organize the structure. They will organize it very well. But don’t say one of the pharmacists, they will come to the surgeon here [Medicine college] or to anyone here and tell them what they will have to do.” F5, Medicine.*

Another issue related to staff was their ability to facilitate IPE sessions. Educators perceived facilitating IPE sessions as something new and unfamiliar to some of the staff. Thus, some expressed their concerns about the readiness and awareness of staff to facilitate IPE sessions involving students from different backgrounds.

*“Some staff may lack experience or training in IPE activities.” F7, Pharmacy.*

*“The other thing, I’m not confident whether instructors will always know how to carry this in a positive way. Because even though it’s mainly discussion led by the students, but the instructor has to play something in it” F1, Applied Medical Sciences.*

## Discussion

Interprofessional education in Arabic speaking countries is still in its early stages. By further investigating and exploring IPE interventions, the body of the evidence in this area will grow and support future implementation. To strengthen evidence in this area, IPE researchers should consider the unique cultures, norms, and education systems of the region. With the involvement of relevant stakeholders, IPE research should explore various aspects related to IPE, including IPE design, delivery, IPE implementation processes and outcome evaluation. The current study targeted important stakeholder groups, namely educators and leaders in healthcare colleges at a Saudi university where IPE implementation is planned. Important insights were gathered from the participants, relying on the SWOC framework to guide the data collection and analysis.

All participants were favorable toward IPE and believed it could benefit students and their future careers in the healthcare system. Participants reported its potential benefits in terms of improving knowledge about other professionals’ roles, communication skills, enhancing efficient patient referral, and improving patient outcomes. The participants’ recognition of IPE’s capacity to cultivate improved interdisciplinary communication and role understanding aligns with established literature highlighting IPE as a crucial mechanism for fostering collaborative practice and ultimately optimizing patient care outcomes (8).

Several points were perceived by participants as strengths that could facilitate the integration of IPE initiatives across the university, including some educators with prior exposure to IPE, the location of certain colleges, and access to an advanced simulation center. In line with the Saudi government’s aspiration to enhance the quality of higher education, academic staff in Saudi universities can pursue postgraduate education in top world-leading universities, qualifying them with the necessary academic research and teaching skills (27). In the context of IPE, participants in this study who studied abroad were exposed to interdisciplinary teaching pedagogies. Such experiences helped equip the faculty with the knowledge and skills needed to implement collaborative learning approaches in local curricula. Staff resistance is among the obstacles that have been documented in relation to IPE implementation process. Among the reasons reported for staff resistance was unfamiliarity with the IPE concept (15). Thus, previous knowledge of IPE would encourage staff acceptance and involvement in IPE integration at the university.

The presence of a simulation center at the university was perceived as a strength that could help with IPE integration. Considering the purpose of IPE, participants in this study believed that IPE should be offered more in simulation experiences. They described that offering IPE in clinical training or exercises that mimic a real hospital ward environment with virtual patients can help achieve IPE outcomes. These views align with the recommendations for introducing IPE in simulation-based learning approaches (28, 29). Some IPE researchers explain that IPE outcomes are achieved progressively; first changes occur at the level of values and attitudes, then at the level of knowledge and responsibilities, and finally at the level of competencies and skills (30). Recommendations by well-known organizations such as Interprofessional Education Collabortive (IPEC) indicate that IPE should be designed and delivered using various approaches, including problem-based learning, team-based learning, case studies, and simulation-based experiences (31). Simulation-based IPE not only enhances students’ motivation and engagement, but more importantly, it provides a safe environment where learners can develop competencies such as effective communication and teamwork skills (32).

Regarding weaknesses, the lack of a university hospital was perceived as a weakness that might slow efforts to incorporate IPE at the university. As reported in the study, some of the colleges are quite distant from each other, so an alternative venue, such as a teaching hospital, was recommended. University hospitals play a significant role in supporting healthcare students’ learning and training. Such institutions will always have the required resources and space for hands-on learning in a collaborative manner (33). While the current solution suggested by some participants is to use the resources available at collaborative hospitals in the region, decision-makers need to consider the need to establish a university hospital.

Another perceived weakness was the high dropout or transfer rate of students from the College of Applied Medical Sciences. Considering the number of participants from applied medical sciences, this information requires further quantitative investigation to accurately determine the transfer or attrition rates of students. In relation to IPE, educators at this college were concerned that learning about other professions in IPE sessions might threaten students’ confidence in their role. This contradicts the available evidence, which shows that IPE increases students’ understanding of their roles and how they fit into the process of providing healthcare for patients (34).

Interacting with other students in the journey of patient care provision helped to strengthen their confidence by knowing how their roles are vital and how they can contribute in the future. However, several criteria should be considered when designing and delivering IPE sessions to avoid potential negative outcomes. Based on contact theory (35), the recommended conditions include equal status for both groups (e.g., having the same number of students from medicine and the same number from nursing), working on common goals, and both groups should have a sense that there is institutional support from the college or university for the session.

Increased collaboration with university staff was perceived as an opportunity. Educators were keen on how working in IPE would encourage staff from different departments to work together. Normally, and due to the traditional way in most universities worldwide, staff from each department work and teach in silos (36). However, institutions with established IPE courses reported the following benefits: enhanced staff collaboration, enhanced research output, and increased quality of education (10, 37).

Educators explained that each year, students in different colleges carry out community-based activities to raise awareness of different topics, such as preventive measures for certain diseases, lifestyle modifications to slow the progression of chronic diseases, or the optimal use of medications. Such activities present great opportunities for IPE for healthcare students from the educators’ perspective. This aligns with published data showing that IPE in community-based services creates meaningful experiences for students (38).

Among the challenges identified by participants was the bureaucratic approval process required before the integration of IPE at the university. According to educators, the approval process requires submitting an application that needs to be approved by different levels of administration at the university. IPE reviewers have called for IPE to be initiated at the highest level in educational institutions. This is referred to as the top-down approach for IPE integration. This approach was recommended by the IPEC and is also known as the institutional approach of implementation, which could address the issue of the bureaucratic approval process (39).

Professional turf or battles were perceived as potential challenges that might be encountered in the process of IPE implementation. Hierarchies and power dynamics in academia can influence learning, research, and institutional growth through intimidation or exclusion, negatively impacting the work environment (40). Territory conflicts between staff from different professions have been reported in studies from different countries. Interestingly, participants from medicine program highlighted that future plans for working in IPE should consider physicians’ egos and ensure that they are given the lead. This finding aligns with the results reported by Alruthia et al., where participants perceived the egos of physicians as an obstacle. The hierarchies in academia might reflect current practices in some medical institutions. In a study by El-Awaisi et al., healthcare professionals mentioned hierarchies as a major barrier to a collaborative environment (41). Comprehensive measures must be taken to cultivate an environment with equal status for all professionals so they can be role models for healthcare students training in hospitals.

The findings of this study constitute the first in the Western region of Saudi Arabia to explore educators’ views regarding IPE. Based on the strengths, weaknesses, opportunities, and challenges identified in this study, several actions can be taken by university leaders planning to initiate IPE to support the implementation process. For instance, educators with prior exposure to IPE can advocate for IPE and help raise awareness of IPE among university staff and students. Rewards and incentives must be considered for staff who contribute to IPE activities to encourage their participation. Community activities at the university can be designed as IPE events to introduce IPE concepts to early year students. Additionally, IPE can be designed and offered as simulation-based experiences in the simulation center targeting advanced-year students. Therefore, efforts are required to ensure that the center is equipped with the required resources to host IPE events. University leaders should consider promoting IPE through collaborative research opportunities.

Similar to other studies in this field, this study has several limitations. The data collected for this study were confined to a single university, which might affect the transferability of this study’s findings. However, the findings reported in this study can be applied to local universities planning to initiate IPE. Few participants from Applied Medical Sciences joined the study. More participants from this college might have had different views than those represented in this study. Future research on IPE in Arabic-speaking countries should explore the enablers and barriers to implementation in universities that have already implemented IPE. To the best of the author’s knowledge, there are two or more universities in Saudi Arabia that have IPE scheduled for healthcare students; however, no published data have been shared from these two universities. The dissemination of staff and student experiences of IPE implementation through publications and conferences is essential for enriching IPE literature in the region. IPE literature in the region needs further investigation on different aspects of IPE implementation process, starting from the first steps of integration and ending with the evaluation of IPE outcomes with the involvement of relevant stakeholders.

## Conclusion

This exploratory study provided insights into the views of educators and leaders on IPE implementation at Taif University. The overwhelming majority of participants had positive views about IPE integration into healthcare programs. Educators with prior exposure to IPE can advocate for IPE and help raise awareness of IPE among university staff and students. Rewards and incentives must be considered for staff who contribute to IPE activities to encourage their participation. Several concerns were highlighted as weaknesses and possible challenges, including beuracractic approval process, lack of a university hospital, professional battles and hierarchies. Improving collaboration between educators from different programs, collaboration with clinicians from hospitals, and IPE community-based activities were perceived as opportunities.

## Data Availability

The raw data supporting the conclusions of this article will be made available by the authors, without undue reservation.
